# Multidrug-resistant pattern of food borne illness associated bacteria isolated from cockroaches in meal serving facilities, Jimma, Ethiopia

**DOI:** 10.4314/ahs.v18i1.6

**Published:** 2018-03

**Authors:** Fithamlak Solomon, Gebre Kibru, Solomon Ali

**Affiliations:** 1 School of Medicine, Wolaita Sodo University, Sodo town, Ethiopia; 2 Department of Medical Laboratory Sciences and Pathology, College of Health Sciences, Jimma University, Jimma city, Ethiopia

**Keywords:** MDR, bacteria, cockroaches, food borne

## Abstract

**Introduction:**

An increase in the emergence and spread of multidrug-resistant (MDR) bacteria in recent years is becoming worrisome. Domestic cockroaches can play a significant role in the dissemination of such bacteria between the environment and human beings. This study aimed at determining anti-microbial resistance pattern of food borne illness associated bacteria identified from cockroaches trapped in restaurants and cafeterias.

**Methods:**

Trapped cockroaches were picked with surgical gloves, sealed in sterile plastic bags and transported to the Microbiology laboratory. Standard microbiological techniques were used to isolate and identify bacteria. Anti-microbial susceptibility testing was done using Kirby Bauer diffusion technique.

**Result:**

A total of five species of food borne illness associated bacteria were detected. Majority (57.1%) of the bacteria were isolated from the gut of cockroaches. More than 89% of the isolates were multi drug resistance (MDR). MDR was higher on gram positive bacteria. *S. aureus* showed 53.3% resistance against oxacillin(MRSA) and 33.3% against vancomycin.

**Conclusion:**

A very high percentage of MDR bacteria was seen in this study. Most of the bacteria tested were isolated from the gut of cockroaches. Potential factors associated with cockroaches that contributed to this high MDR rate of the isolates should be investigated in future.

## Introduction

Antibiotic resistance is a major public health global concern, with fears expressed that we shortly could run out of antibiotics. Many research articles on antibiotic resistance start with a statement about the threat drug resistance poses towards public health.^1^

Bacterial drug resistance is a worldwide problem that is aggravated by the diminishing number of new antimicrobial drugs in the pharmaceutical pipeline.^2^ The effectiveness of currently available antibiotics is decreasing due to the increasing number of resistant strains. The available therapeutic options for antibiotic-resistant organisms are also severely limited, as these organisms frequently display a multi-drug resistant (MDR) phenotype.^3^ Antimicrobial resistant bacteria can be transferred across by human, animal and insect vectors.^4^ Pests that develop in decaying organic material may transmit anti-microbial drug resistant bacteria from the manure of animals and other decaying organic substrates to residential settings.^5^ Different arthropods such as cockroaches can move freely between human/animal wastes and may play a significant role in the dissemination of drug resistant bacteria to the environment and human beings.^6^

Several studies confirmed the potential of cockroaches in carrying drug resistance pathogens through their cuticle and guts. For instance, food borne illness associated bacteria isolated from cockroaches in Ethiopia^7^, India^8^, Brazil^9^, Taiwan^10^ and Libya^11^ were resistant against tested anti-microbial drugs. In these studies, about 30–100% *S.aureus* and 14%–100% *Enterobacteriaceae* isolates were reported to be resistant against ampicillin, streptomycin, tetracycline, erythromycin or trimethoprim-sulfamethoxazole. 8,9,10,11.

All those available documents indicated that there is a great variability in anti-microbial resistance of bacteria isolates with respect to cockroaches geographical location and foraging habitat. Hence, this study was designed to determine the rate of anti-microbial resistance as well as MDR pattern of food borne illness associated bacteria isolated from cockroaches foraging in food and drinking handling establishments at Jimma town, Ethiopia.

## Materials and methods

### Study design

Cross-sectional study was done from May l to September 30, 2012 from cockroaches which were collected at restaurants and cafeterias in Jimma town. A cockroach trapping was made using Hoy-Hoy roach sticky traps.^12^ The traps were placed in areas which were hard to reach, dark and crammed corners at different sections of food handling establishments. Sticky traps containing cockroaches were picked with sterile surgical gloves, sealed in sterile plastic bags.^13^ and transported to Jimma University Microbiology Laboratory by cold chain system for isolation of bacteria.

### Microbiological sample preparation

A total of 1140 trapped cockroaches were picked from the sticky traps by sterile forceps and pooled in batches (i.e. ten cockroaches pooled as one sample). The pooled cockroaches were anesthetized in a sterile jar by using chloroform soaked cotton.^7^ The immobilized cockroaches were placed in 5 ml sterile physiological saline (0.85%) and placed in a shaker for two minutes to dislodge bacteria from its body surfaces. Then, the wash was taken as external body homogenate sample to isolate bacteria.^8^ Subsequently, dislodged cockroaches were soaked in 90% ethanol for five minutes and dried to decontaminate their external surfaces. After that, they were rewashed with sterile physiological saline (0.85% NaCl) in order to remove traces of ethanol. Then, the cockroaches' alimentary tract was aseptically dissected out using autoclave-sterilized entomological dissecting needles under a dissecting microscope. The instruments were dipped in ethanol and flamed between dissections. The excised gut was then homogenized in 5 ml sterile normal saline and the homogenates were used as gut sample to isolate bacteria.^8^

### Isolation and identification of bacterial

One ml of each external and gut homogenate samples were suspended separately into 9 ml sterile bottles containing buffered peptone water (Oxoid, Hampshire, UK) and incubated at 37°C for 18–24h. The pre-enriched yield solutions were then separately inoculated on MacConkey, Xylose lysine deoxycholate agar (XLD), Mannitol salt agar (MSA) and Polymyxin-B egg yolk Mannitol Bacillus cereus agar (PEMBA). Rappaport-Vassiladis (RV) broth was also used as a primary enrichment medium for the identification of *Salmonella* and *Shigella*. Then, enrichment broths and agar plates were incubated at 37°C for 24h. The bacteria growth on the agar media were identified by colonial morphology, Gram-staining and a battery of biochemical tests (oxidase, catalase, simmon citrate, indoleproduction, urease, motility, coagulase, methyl red-Voges Proskaeur(MR-VP), lysine decarboxylase (LDC), Klingler's iron agar (KIA), mannitol fermentation, gas and H2S production).^14^,^15^ Sero-grouping of *Salmonella* spp was done by slide agglutination technique using poly O (A-I) and monovalent (O2, O3, O4, O5, O6, O7, O8, O9, O15 and Vi) antigens for identification of *Salmonella* serogroups, A-E (Difco, Detroit, USA). Similarly, *Shigella* sero grouping was done using known polyvalent *Shigella* antisera A, B, C and D (Remel, Europe Ltd, UK). Physiological saline was used in the test as a negative control.^16^

### In vitro drug susceptibility testing

Anti-microbial susceptibility testing was done on Mueller-Hinton agar (with 5% sheep blood agar for B. cereus) (Oxoid, Hampshire, UK) using the standardized Kirby Bauer diffusion technique as per the recommendations of Clinical Laboratory Standard Institute.^17^ The turbidity of the bacterial suspension was adjusted to the density of a McFarland 0.5 in order to standardize the inoculums size. For susceptibility testing, the following antimicrobial disks with their respective concentration were used: Ampicillin (Amp,30µg); Cephalothin (Kf,30µg); Chloramphenicol (Caf,30µg); Gentamicin(Cn,10µg); Ciprofloxacin (Cip,5µg); Polymyxin B (Pol,30µg); Streptomycin (Str,10µg); Clindamycin (Cli,2µg); Erythromycin (Ert,15µg); Norfloxacin (Nor,10µg); Oxacillin (Ox,1µg); Ceftriaxone(Cro,30µg); Penicillin G (Pen,10µg); Tetracycline (Tet,30µg);Trimetoprim-Sulfamethoxazole (Sxt, 25µg) and Vancomycin (Van,30µg). These drugs were selected based on local availability, clinical efficiency, literatures and CLSI guideline.^17^ In this study, MDR is defined as resistance of bacteria against two or more classes of antibiotics (CDC)^18^. Furthermore, the quality of each disk was monitored by using standard *S. aureus* (ATCC 6538) and *E. coli* (ATCC 25922) reference strain.

### Data analysis

Data was entered and checked for completeness and exported to SPSS version 16 for analysis. Summary statistics such as frequencies and percentages were computed. Descriptive statistics, frequencies and percentages was used to determine drug resistance isolates and MDR pattern.

### Ethics

Approval and permission for the study was obtained from the Institutional Review Board (IRB) of the College of Health Sciences, Jimma University. A support letter for each cafeteria and restaurant was written by the Jimma municipality. Consent was also obtained from the owner of each cafeteria and restaurant.

## Results

**Figure 1:**
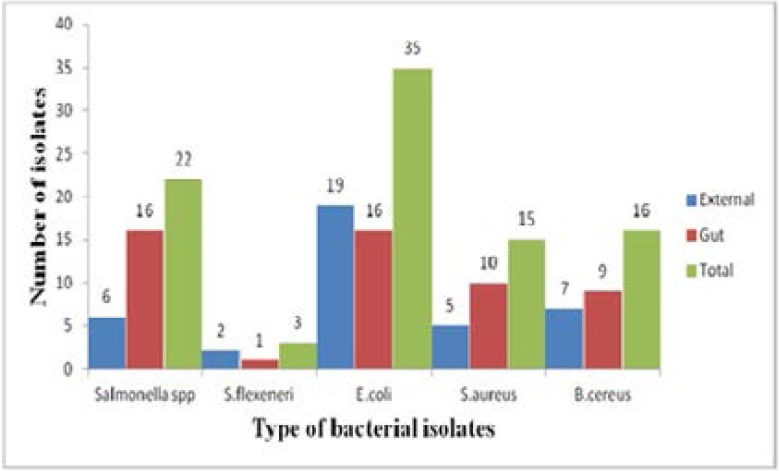
Frequency of species of food borne bacteria pathogens isolated from Blattella germanica cockroaches

A total of 91 Gram negative and Gram positive bacteria were isolated from the gut and body surfaces of cockroaches. Five bacteria species which are associated with food borne illness (*Sallmonella* spp., *Shigella flexneri, E. coli*, *S.aureus* and *B. cereus* ) were isolated. Most (57.1%) of the isolates were recovered from the gut of cockroaches (Figure 1).

**Figure 2:**
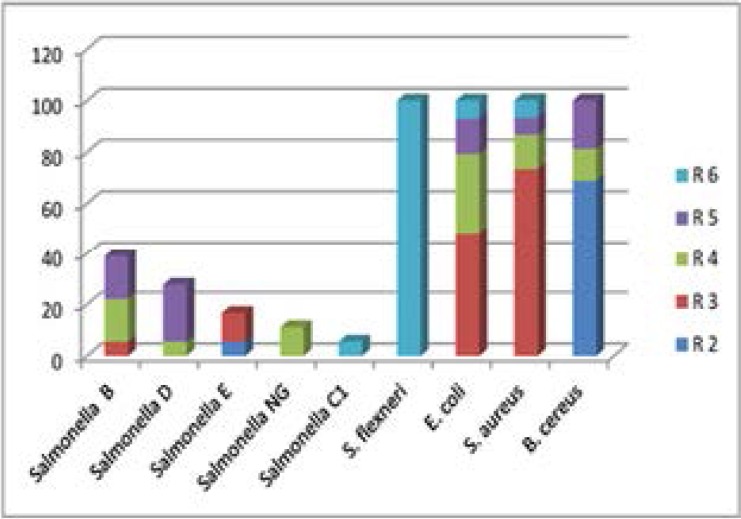
percentage of MDR pattern of Foodborne bacterial pathogens isolated from cockroaches at meal serving facilities in Jimma town. Where R2= resistance against any two of the antibiotic classes of Penicillins, Cephalosporines, Aminoglycosides, Tetracycline, and Macrolides; R3-R6 =Resistance against any three, four, five or six of the antibiotic classes of Penicillins, Cephalosporines, Aminoglycosides, Tetracycline, Macrolides , Chloramphenicol,, Sulfonamides,Vancomycine and Quinolens.

The overall MDR rate was 81(89.0%). All isolated *S. aureus* and *B.cerus* were MDR; resistant against at least two classes of antibiotics (figure 2). The overall prevalence of MDR among Gram negative bacteria isolates was 50(83.3%). Similarly all isolated *Shigella flexneri* were MDR (figure 2). About, 18(81.8%) of *Salmonella* spp. were also MDR. Among *salmonella* sero-types *Salmonella B* and *Salmonella D* were resistant against four or more classes of antibiotics. More than four fifth 29(82.9%) of *E. coli* were resistant against at least two classes of drugs (figure 2).

All (n=31) Gram positive bacteria (*S.aureus* and *B. cereus*) were resistant against penicillin G and 77% of them were resistant against oxacillin and 25.8% to vancomycin. On the other hand, both isolates were relatively sensitive for chloramphenicol (Table 1).

**Table 1:** Anti-microbial resistance pattern of *S. aureus* and *B. cereus* identified from cockroaches at Jimma town (May 1 to September 30, 2012), Jimma, SouthWest Ethiopia.

Drugs	*S. aureus* (n=15)			*B. cereus* (n=16)			Total (n=31)		
	S No (%)	I No(%)	R No (%)	S No (%)	I No(%)	R No (%)	S No (%)	I No(%)	R No (%)
**NOR**	13(86.7)	2(13.3)	0(0)	13(81.3)	0(0)	3(18.8)	26(83.9)	2(6.5)	3(9.7)
**OX**	7(46.7)	-	8(53.3)	0(0)	-	16(100)	7(22.6)	-	24(77.4)
**VAN**	10(66.7)	-	5(33.3)	13(81.3)	-	3(18.8)	23(74.2)	-	8(25.8)
**PEN**	0(0)	0(0)	15(100)	0(0)	0(0)	16(100)	0	0(0)	31(100)
**CLI**	9(60)	0(0)	6(40)	9(56.3)	1(6.3)	6(37.5)	18(58.1)	1(3.2)	12(38.7)
**ERT**	5(33.3)	2(13.3)	8(53.3)	12(75)	0(0)	4(25)	17(54.8)	2(6.5)	12(38.7)
**TET**	7(46.7)	1(6.7)	7(46.7)	9(56.3)	4(25)	3(18.8)	16(51.6)	5(16.1)	10(32.3)
**CIP**	8(53.3)	4(26.7)	3(20)	13(81.3)	0(0)	3(18.8)	21(67.7)	4(12.9)	6(19.4)
**CAF**	15(100)	0(0)	0(0)	16(100)	0(0)	0(0)	31(100)	0(0)	0(0)
**KF**	6(40)	2(13.3)	7(46.7)	7(43.8)	3(18.8)	6(37.5)	13(41.9)	5(16.1)	13(41.9)
**STR**	8(53.3)	3(20)	4(26.7)	6(37.4)	3(18.8)	7(43.8)	14(45.2)	6(19.4)	11(35.5)

NOR - Norfloxacin, OX - Oxacillin, VAN - Vancomycin, PEN - Penicillin G, CLI - Clindamycin, ERT - Erythromycin, TET - Tetracycline, CIP - Ciprofloxacin, CAF - Chloramphenicol, KF - Cephalothin, STR - Streptomycin, S - Sensitive, R - Resistance, I-Intermediate

The anti-microbial susceptibility profile of Gram negative bacterial isolates (n=60) showed that, highest (81.6%) resistance was seen against ampicillin followed by 73.3% against tetracycline and cephalosporin each. Low resistance of Gram negative bacteria were seen against gentamicin (5%) and ciprofloxacin (11.7%). On the other hand, all Gram negative bacteria were sensitive to polymixin B (Table 2).

**Table 2:** Anti-microbial resistance pattern of gram negative food borne associated illness bacterial isolated from cockroaches at Jimma Town (May 1 to September 30, 2012), Jimma, SouthWest Ethiopia.

Drugs	*Salmonella spp* (n=22)			*Shigella flexneri* (n =3)			*E. coli* (n=35)			Total (n=60)		
	S No (%)	I No (%)	R No (%)	S No(%)	I No (%)	R No(%)	S No (%)	I No (%)	R No (%)	S No (%)	I No (%)	R No (%)
**TET**	7(31.8)	1(4.5)	14(63.6)	0(0)	0(0)	3(100)	8(22.9)	0(0)	27(77.1)	15(25)	1(1.7)	44(73.3)
**CIP**	12(54.5)	5(22.7)	5(22.7)	0(0)	0(0)	3(100)	27(77.1)	3(8.6)	5(14.3)	39(65)	8(13.3)	13(21.7)
**CAF**	12(54.5)	1(4.5)	9(40.9)	0(0)	0(0)	3(100)	25(71.4)	4(11.4)	6(17.1)	37(61.7)	5(8.3)	18(30)
**KF**	6(27.3)	1(4.5)	15(68.2)	0(0)	0(0)	3(100)	9(25.7)	0(0)	26(74.3)	15(25)	1(1.7)	44(73.3)
**STR**	8(36.3)	2(9.1)	12(54.5)	0(0)	0(0)	3(100)	16(45.7)	5(14.3)	14(40)	24(40)	7(11.7)	29(48.3)
**CN**	22(100)	0(0)	0(0)	3(100)	0(0)	0(0)	29(82.9)	3(8.6)	3(8.6)	54(90)	3(5)	3(5)
**POL**	22(100)	0(0)	0(0)	3(100)	0(0)	0(0)	35(100)	0(0)	0(0)	60(100)	0(0)	0(0)
**TMP-SXT**	5(22.7)	1(4.5)	16(72.7)	0(0)	0(0)	3(100)	19(54.3)	3(8.6)	13(37.1)	24(40)	4(6.7)	32(53.3)
**CRO**	14(63.6)	1(4.5)	7(31.8)	3(100)	0(0)	0(0)	33(94.3)	2(5.7)	0(0)	50(83.3)	3(5)	7(11.7)
**AMP**	9(40.9)	0(0)	13(59.1)	0(0)	0(0)	3(100)	0(0)	2(5.7)	33(94.3)	9(15)	2(3.3)	49(81.6)

TET-Tetracycline, CIP-Ciprofloxacin, CAF-Chloramphenicol, KF-Cephalothin, STR-Streptomycin, CN-Gentamicin, POL-PolymyxinB, TMP-SXT-Trimethoprim-Sulfamethoxazole, CRO-Ceftriaxone, AMP-Ampicillin, S-Sensitive, I-Intermediate, R-Resistan

## Discussion

The antibiotic resistance and MDR pattern of the bacteria isolates observed in this study is very worrisome. Eighty nine percent of isolated bacteria were resistant to at least two classes of antibiotics (MDR). On top of this, 57.1% of isolated bacteria were recovered from the gut of cockroaches. This finding is an interesting finding in a way that MDR bacteria might be well protected from the action of disinfectants which are applied during routine cleaning.. Furthermore, there is a possibility for these bacteria to replicate in the gut of cockroaches and be released to the environment during feeding or defecation. If other conditions for disease occurrence were fulfilled, like susceptible host and route of entry, such bacteria are highly likely to cause severe disease which may be difficult to treat.^19^

All isolated food borne illness associated Gram positive bacteria were MDR. The overall rate of MDR *S.aureus* seen in this study is much higher than the 72% MDR bacteria identified from nasal cavity of hospitalized patients which was reported before in the same area.^20^ In comparison with other similar studies done on bacteria isolated from cockroaches foraging at hospital environment in Ethiopia, the MDR rate observed in this study was low.^7^ More than half and one third of *S.aureus* isolates were resistant against methicillin and vancomycin antibiotics respectively. The emergence of vancomycin resistance *S.aureus* is becoming a serious public health concern. It jeopardizes the available antibiotic reserve option against these bacteria.^21^ In this regard, cockroaches might also be considered as one of the potential threats by harboring vancomycin resistance *S. aureus*. Previous report on *S.aureus* isolated from nasal cavity of hospitalized patients in the same area indicated low prevalence of vancomycin resistance *S.aureus*.^20^ To explain this difference, it seems the gut of cockroaches might be favoring the survival of vancomycin resistance *S.aureus*. Furthermore, the possibility of drug resistance gene exchange through horizontal gene transfer mechanism among strains inside the gut could also be considered.

*S. aureus* was also resistant against most of the antimicrobial drugs tested except norfloxacin and chloramphenicol with a range of resistance that varies from 20% to 100%. The observed 100% penicillin G resistance in the current study is consistent with 100% and 97.2% resistance rate reported in Ethiopia before.^7^,^22^

All *B. cereus* isolates identified from cockroaches in this study were oxacillin and penicillin G resistant. This might be associated with drug resistance character of *B. cereus* which is usually attributable to β-lactamase production. The prevalence of vancomycin resistant *B. cereus* in our study corresponds with 20.8% and 20% resistance which was reported in Botswana^23^ and Nigeria^24^ respectively. The drug of choice for most *B. cereus* related infection in humans is vancomycin and ciprofloxacin. Towards this end identification of vancomycin and ciprofloxacin resistant *B. cereus* from cockroaches which have free wandering movement in food handling establishments can pose considerable threat. The presence of interchangeable plasmid transferring genes in this bacterium makes it possible for the transfer of antibiotic resistance genes between bacteria and also across the insects.^25^

The drug resistance pattern of *Salmonella* spp to the majority of tested antimicrobials was lower than previous reports made on similar study in Addis Ababa, Ethiopia.^7^ This could be explained by the fact that isolates in previous studies were obtained from cockroach's trapped in hospital environments where rate of drug resistance bacteria is supposed to be higher and cockroaches may harbor such bacteria as they crawl in these areas. However, the MDR rate of *salmonella* species seen in this study was very high. Given that, the bacteria are truly pathogenic, the potential treat is high if these bacteria get access to susceptible host or community at large.

In this study, the observed MDR rate of *E. coli* was also very high. Though the bacteria is considered as normal flora of the gut it needs equal attention. Acquisition of plasmid through horizontal gene transfer mechanism can transduce the normal flora *E. coli* bacteria to virulent. Should these MDR seen was associated with plasmids, it might have the potential to transduce other susceptible *E. coli* strains which reside in the cockroaches.

Ampicillin resistant *E.coli* isolates, 33(94.3%), in the current study is comparable with 100% resistance rate reported in Addis Ababa.^21^ But, our finding is slightly higher compared with resistance rate of 77.4% in Brazil^9^ and 84.6% in Taiwan^26^ It is also observed here that 14.3% of *E. coli* were resistant for ciprofloxacin. This finding was higher compared with 4% ciprofloxacin resistance reported in Taiwan.^27^

Like any other studies, this study had its own limitations and should be interpreted with caution. Genotyping of the isolates to depict if there was multiplication and/or plasmid acquisition inside the gut of cockroaches was not done. As a result comparison of the genetic profile of MDR isolates to depict replication and/or horizontal gene transfer among isolated strains was hampered. Extended spectrum beta lactamase (ESBL) production of isolated *E. coli* was not done due to logistics constraints. Apart from these, we believe this study pointed out valuable findings and perspectives for future studies.

## Conclusion

A very high percentage of MDR bacteria was seen in this study. Majority of the bacteria tested were isolated from the gut of the cockroaches. Further study on genotyping of bacteria isolated from gut of cockroaches is recommended to depict the potential multiplication and acquisition of mobile genes in the gut of the cockroaches. Our data has indicated the possibility of considering cockroaches in future efforts dealing with drug resistance. From public health point of view, it is important to control cockroaches foraging in public food and drinking establishments due to the potential risk of transmitting these MDR bacteria to susceptible host.
